# Swelling and Diffusion in Polymerized Ionic Liquids-Based Hydrogels

**DOI:** 10.3390/polym13111834

**Published:** 2021-06-01

**Authors:** Ann Jastram, Tobias Lindner, Christian Luebbert, Gabriele Sadowski, Udo Kragl

**Affiliations:** 1 Institute of Chemistry, Industrial Chemistry, University of Rostock, Albert-Einstein-Str. 3a, 18059 Rostock, Germany; ann.jastram@uni-rostock.de; 2 Core Facility Multimodal Small Animal Imaging, Rostock University Medical Center, Schillingallee 69a, 18057 Rostock, Germany; tobias.lindner@med.uni-rostock.de; 3 Amofor GmbH, Otto-Hahn-Str. 15, 44227 Dortmund, Germany; luebbert@amofor.de; 4 Laboratory of Thermodynamics, Department of Biochemical and Chemical Engineering, TU Dortmund University, Emil-Figge-Str. 70, 44227 Dortmund, Germany; gabriele.sadowski@tu-dortmund.de; 5 Department Life, Light & Matter, Faculty for Interdisciplinary Research, University of Rostock, Albert-Einstein-Str. 25, 18059 Rostock, Germany

**Keywords:** polymerized ionic liquids, hydrogels, swelling, diffusion, sorption experiments, magnetic resonance imaging

## Abstract

Hydrogels are one of the emerging classes of materials in current research. Besides their numerous applications in the medical sector as a drug delivery system or in tissue replacement, they are also suitable as irrigation components or as immobilization matrices in catalysis. For optimal application of these compounds, knowledge of the swelling properties and the diffusion mechanisms occurring in the gels is mandatory. This study is focused on hydrogels synthesized by radical polymerization of imidazolium-based ionic liquids. Both the swelling and diffusion behavior of these hydrogels were investigated via gravimetric swelling as well as sorption experiments implemented in water, ethanol, *n*-heptane, and tetrahydrofuran. In water and ethanol, strong swelling was observed while the transport mechanism deviated from Fickian-type behavior. By varying the counterion and the chain length of the cation, their influences on the processes were observed. The calculation of the diffusion coefficients delivered values in the range of 10^−10^ to 10^−12^ m^2^ s^−1^. The gravimetric results were supported by apparent diffusion coefficients measured through diffusion-weighted magnetic resonance imaging. A visualization of the water diffusion front within the hydrogel should help to further elucidate the diffusion processes in the imidazolium-based hydrogels.

## 1. Introduction

Hydrogels are formed by the linkage of hydrophilic monomers resulting in three-dimensional networks having a number of favorable properties. Mainly depending on the type of monomer and to a lesser extent on the crosslinking degree, these gels may absorb, store, and release up to 90% *w*/*w* of water combined with a strong swelling and de-swelling [1,2]. However, due to entanglements and chemical bonds within the chain, no dissolution of the polymer chains is observed in an aqueous environment, so the swelling gels maintain their 3D shape despite the increase in volume [3,4]. Adaptable properties like surface hardness, temperature resistance, flexibility, or stiffness combined with inherent ones like biocompatibility, biodegradability, and the lack of toxicity make them interesting materials that gives them significant potential in many different fields. The most popular ones are the medical and pharmaceutical sectors exploring and applying hydrogels as materials for contact lenses [5], implants [6], stent coatings [1], drug delivery systems [7,8,9], and in tissue engineering [10,11]. Moreover, these polymers also have a great potential as immobilization matrices to improve catalyst recovery, which is particularly recognized in homogeneous processes. In 2015, we reported a successful encapsulation of a quinine-based organocatalyst into imidazolium-based polymerized ionic liquids (pILs). Its utilization for the asymmetric nitroaldol (Henry) reaction showed a reduced reaction rate compared to the homogenously soluble one [12]. We also showed the encapsulation of lipase B from *Candida antarctica* in these hydrogels, resulting in a reduced reaction rate compared to commercially available lipases immobilized on carriers [13]. In both cases, the structure of the matrix has to be considered due to its impact on the diffusion behavior. Generally, the diffusion of molecules in hydrogels is hindered. It depends on the crosslinker density, the hydrodynamic radius of the diffusing species inside the network, as well as the interactions between the diffusing species and the polymer network. Especially considering the complexity of the hydrogel network as a disordered arrangement of polymer chains having multifunctional junctions, loops, physical entanglements, and unreacted end groups is critical. The interaction of all these parameters is mandatory to understand the diffusion behavior [14,15,16,17]. Thus, knowledge about solute diffusion within the hydrogels is necessary for practical applications in the medical, pharmaceutical, biological, or environmental sectors as well as for the targeted production of substances for specific applications. In the last decades, many different methods for diffusion investigation in polymer networks have been developed. Some of the methods are mainly informative at a macroscopic level as they refer to measurements outside the gel not relating to the properties of the solvent inside the network or the mechanisms of the diffusion-controlling processes. Release experiments are one of the most utilized methods belonging to this group [18,19]. Other techniques try to examine the processes inside the network, e.g., confocal 1D-Raman spectroscopy [20]. Furthermore, pulsed-field gradient magnetic resonance techniques with strong field gradients are often reported as an efficient tool to investigate processes in gels [21]. A representative of this group is the nuclear magnetic resonance imaging (MRI) that is usually found in medicine and biology for cross-sectional or 3D images of living organisms and solid materials. For hydrogels, it has been used to provide information about possible abnormal diffusion processes [22], control drug release [23], or to check hydrogel fixation for tissue engineering [24]. In this study, we used MRI images to examine the nature of the observed diffusion processes on a spatially resolved level for hydrogels based on polymerized ionic liquids. Moreover, the diffusion coefficients and the diffusion types were determined by the recording of sorption curves as well as measuring the mass uptake of the hydrogels after soaking them in a solvent for a certain time interval. Therefore, we used three methods with different focal points on an internal and external level. The obtained information should help to further elucidate and better understand the diffusion processes in the imidazolium-based hydrogels.

## 2. Materials and Methods

### 2.1. Chemicals

Rotiphorese Gel B (MBAA) (2% *w*/*w N,N′*-methylenebis(acrylamide), Carl Roth, Karlsruhe, Germany), *N,N,N′,N′*-tetramethylethylenediamine (TEMED) (≥99.5%; Sigma Aldrich, St. Louis, MO, USA), ammonium persulfate (APS) (98%; Acros Organics, Fair Lawn, NJ, USA), 1-vinylimidazole (≥99%; Alfa Aesar, Haverhill, MA, USA), bromoethane (98%; Alfa Aesar), 1-chlorobutane (≥99%; Acros Organics), 1 bromobutane (99%; Sigma Aldrich), ethanol (EtOH) (≥99.8%), *n*-heptane (*n*-Hep) (≥99.5%), and tetrahydrofuran (THF) (≥99.8%) were used in this study. Additionally, ultrapure water was used throughout the study.

### 2.2. Synthesis of Polymerizable Monomers

For this study, 1-vinyl-3-ethyl-imidazolium bromide (VEImBr), 1-vinyl-3-butyl-imidazolium chloride (VBImCl), and 1-vinyl-3-butyl-imidazolium bromide (VBImBr) were prepared according to published procedures [25,26,27,28]. The recorded NMR spectra can be found in the supplementary materials (Figures S1–S3).

VEImBr: 1H NMR [300 MHz, DMSO-d6, δ/ppm relative to tetramethylsilane (TMS)]: 9.64 (s, 1H, N=CH–N), 8.24 (s, 1H, N–CH=CH–N), 7.98 (s, 1H, N–CH=CH–N), 7.32 (dd, J = 15.77 Hz, J = 8.82 Hz, 1H, N–CH=CH_2_), 5.99 (dd, J = 15.73 Hz, J = 2.37 Hz, 1H, N–CH=CH_2_), 5.41 (dd, J = 8.78 Hz, J = 2.29 Hz, 1H, N–CH=CH_2_), 4.24 (q, J = 7.41 Hz, 2H, ethyl-α-CH_2_), 1.45 (t, J = 14.64 Hz, 3H, ethyl-β-CH_3_).

VBImCl: 1H NMR [300 MHz, DMSO-d6, δ/ppm relative to tetramethylsilane (TMS)]: 9.93 (s, 1H, N=CH–N), 8.31 (s, 1H, N–CH=CH–N), 8.01 (s, 1H, N–CH=CH–N), 7.37 (dd, J = 15.75 Hz, J = 8.80 Hz, 1H, N–CH=CH2), 6.04 (dd, J = 15.63 Hz, J = 2.37 Hz, 1H, N–CH=CH_2_), 5.40 (dd, J = 8.78 Hz, J = 2.29 Hz, 1H, N–CH=CH_2_), 4.23 (t, J = 7.18 Hz, 2H, butyl-α-CH_2_), 1.82 (q, 2H, butyl-β-CH_2_), 1.28 (sxt, 2H, butyl-γ-CH_2_), 0.90 (t, 3H, butyl-δ-CH_3_).

VBImBr: 1H NMR [300 MHz, DMSO-d6, δ/ppm relative to tetramethylsilane (TMS)]: 9.64 (s, 1H, N=CH–N), 8.24 (s, 1H, N–CH=CH-N), 7.98 (s, 1H, N–CH=CH–N), 7.32 (dd, J = 15.58 Hz, J = 8.78 Hz, 1H, N–CH=CH_2_), 5.99 (dd, J = 15.71 Hz, J = 2.39 Hz, 1H, N–CH=CH_2_), 5.42 (dd, J = 8.76 Hz, J = 2.29 Hz, 1H, N–CH=CH_2_), 4.22 (t, J = 7.27 Hz, 2H, butyl-α-CH_2_), 1.81 (q, 2H, butyl-β-CH_2_), 1.28 (sxt, 2H, butyl-γ-CH_2_), 0.91 (t, 3H, butyl-δ-CH_3_).

### 2.3. General Procedure for Hydrogel Synthesis

**Scheme 1:**
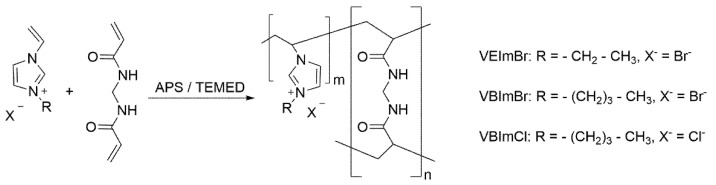
Synthesis of pILs-based hydrogels via radical polymerization with MBAA as a crosslinker.

**Figure 1:**
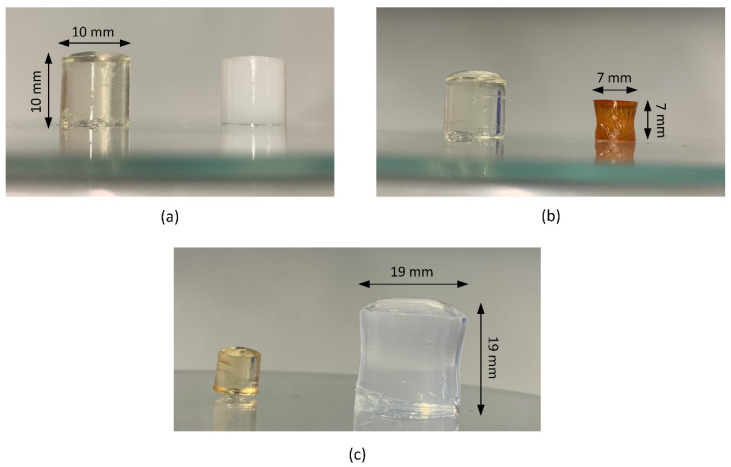
Cylindrical hydrogels (**a**) with different MBAA contents (left: 2 mol%; right: 5 mol%), (**b**) at different drying stages (left: without drying; right: after 5 d at 60 °C; 2 mol% MBAA) and (**c**) after swelling in water (left: unswollen; right: swollen for 24 h at 30 °C).

For the gravimetric swelling and the MRI experiments, the vinylimidazolium-based hydrogels were synthesized in a standardized procedure at room temperature (22 ± 2 °C) as reported earlier [29]. To modulate a crosslinker content of 2 mol%, the respective amount of IL monomer (0.300 g VEImBr, 0.341 g VBImBr, 0.276 g VBImCl) was dissolved in 432 µL of ultrapure water and 232 µL of crosslinker solution (MBAA, 2% *w*/*w*) was added. Radical polymerization (Scheme 1) was initiated by adding 30 µL of ammonium persulfate solution (APS, 10% *w*/*w*) and 6 µL of TEMED immediately before mixing the solution thoroughly for 10 s. It was then allowed to gel for 30–50 min into cylindrical shaped molds (10 mm diameter, 10 mm height). After removal of the gels, they were stored in a compartment dryer at 60 °C for 5 d with subsequent drying in a desiccator until weight constancy was established. For illustrative purposes, cylindrical hydrogel samples with different MBAA contents and at different drying as well as swelling levels are visualized in Figure 1.

For the gravimetric sorption experiments, four times of the described preparation quantity was used and allowed to gel for 20 min in a square mold (40 mm × 40 mm). After removal and cutting of the gel layers into cuboidal pieces, the gels were first dried for 5 d at 60 °C in a compartment dryer followed by 16 h in a high vacuum. The synthesis of hydrogels having crosslinker contents of 3 mol% and 5 mol% was realized by varying the volumes of water and MBAA solution (Table 1). After drying, all samples were stored under an argon atmosphere. Within this work, all hydrogels are named with the prefix “poly”.

### 2.4. Gravimetric Swelling Experiments

The solvent uptake was measured gravimetrically by weighing the mass of the (swollen) gels as a function of time. When reaching the equilibrium state, the mass remained constant, and the measurement was finished. The sample sizes varied between 6.31 mm to 6.76 mm in diameter and 6.51 mm to 7.50 mm in height. After determining the dry mass of the samples, the gels were placed in a strainer that was doused in a crystallization dish containing 900 mL of the water, EtOH, THF or n-Hep and allowed to soak at 30 ± 1 °C. The strainers were taken out at monitored time intervals. Both the strainers and the gels were carefully dabbed with a lint-free paper towel to eliminate surface-bond solvent. It was then weighed and returned to the solution again. These dynamic swelling studies were performed in triplicate to investigate the swelling behavior and the mechanism of solvent diffusion in the hydrogel.

### 2.5. Calculations from Gravimetric Swelling Experiments

The experimental equilibrium swelling (S_eq, exp_) of the hydrogels was calculated from the data by the following term

(1) $$S_{\mathrm{eq},\;\exp.}=\frac{M_{\infty }-M_{0}}{M_{0}}$$

with M_∞_ being the mass of the absorbed water in the equilibrium state and M_0_ being the initial dry mass at the time t = 0.

For a quantitative representation of the absorbed water amount, the equilibrium water content (EWC) was calculated using the following

(2) $$\mathrm{EWC}=\frac{M_{\infty }-M_{0}}{M_{\infty }}$$

Additionally, the kinetics of polymer swelling were investigated in more detail. The process can be described using the following second-order relation

(3) $$\frac{t}{S}=A+B\cdot t$$

where B = 1/Seq corresponds with the reciprocal of the equilibrium swelling, A = 1/k_s_∙Seq^2^ includes the inverse of the initial swelling rate (dS/dt)_0_ and the swelling rate constant k_s_. The theoretical values of the equilibrium swelling, the initial swelling rates, and the swelling rate constants were calculated from the slope and the y-intercepts of the linear regression of the experimental data [30].

The type of diffusion was determined by the following expression

(4) $$\frac{M_{t}}{M_{\infty }}=k\cdot t^{n}$$

where M_t_ is the mass of the absorbed water at the time t, k is a characteristic constant of the system, and the exponent n characterizes the mode of the solute transport. By plotting ln(M_t_/M_∞_) as a function of ln(t), n results from the slope and k from the axis intercept [31,32]. The diffusion coefficient can be deduced from the dynamic part of the swelling behavior. For this purpose, the approximation of Fick’s equation for the diffusion of the swelling agent was used

(5) $$\frac{M_{t}}{M_{\infty }}=4\cdot {\left(\frac{D\cdot t}{\pi \cdot l^{2}}\right)}^{0.5}$$

where D is the diffusion coefficient and l the diameter of the hydrogel cylinder. D is calculated using the help of the following term

(6) $$D={\left(a\cdot \frac{{\left(\pi \cdot l^{2}\right)}^{0.5}}{4}\right)}^{2}$$

including a as the linear area of the slope taken from the plot of M_t_/M_∞_ against the square root of the time [31,33].

### 2.6. Diffusion Coefficients from Interval Sorption Experiments

A flow-through setup with continuous data recording was used to perform these experiments. The setup has already been described in 2005 by Krüger and Sadowski [34]. Its centerpiece is a magnetic suspension balance that allows for investigating sorption in samples weighing up to 30 g. Placing the balance outside the measuring cell ensures long-term stability as well as a high accuracy, resulting in a reproducibility of ±0.3 mg. The dimensions of the completely dried hydrogel cuboids were noted before they were put inside a glass bucket connected to the balance. After assembling the apparatus, the measurement cell was evacuated using a turbomolecular vacuum pump (pressure < 10^−5^ mbar). After reaching a constant mass, the inlet valve was opened and the solvent vapor was led from the vaporizer into the cell with a constant flow resulting in predefined solvent partial pressures of 25%, 50%, and 75%. Each measurement started with a pressure jump from 0 mbar up to a pressure that matched 25% relative humidity (RH). The solvent vapor pressure was controlled by the valve at the outlet of the cell and measured by means of a capacitive pressure sensor. Just when the sample mass became constant again, the humidity in the cell was set to the next solvent partial pressure. The temperature control of the housing was realized by an air thermostat, solvent evaporation was performed in a double-walled glass vessel heated by means of a liquid thermostat. The temperatures were checked by PT100 sensors with a correctness of ±0.05 K. All measurements were performed at 30 ± 1 °C.

D was obtained from the data by using Fick’s second law of diffusion. Under the assumption of a constant D, Crank solved the law for a free-standing film experiencing an abrupt increase in external concentration on both sides [35].

(7) $$\frac{m_{t}}{m_{\infty }}=1-\overset{\infty }{\underset{n=0}{\sum }}\frac{8}{{\left(2n+1\right)}^{2}\pi^{2}}\exp\left\{-\frac{D{\left(2n+1\right)}^{2}\pi^{2}t}{d^{2}}\right\}$$

If the samples are subjected to higher surface concentrations on only one side, the film thickness d that is measured at the beginning of this sorption interval must be squared in the equation. D is assumed as the mean diffusion coefficient, which was adjusted to the respective sorption curve [34].

### 2.7. Diffusion Weighted Magnetic Resonance Imaging (DW-MRI)

All hydrogels were conscientiously dried as described above. The DW-MRI measurements were carried out at 21.5 ± 2 °C with the help of a BioSpec 70/30 from Bruker (Karlsruhe, Deutschland) having a field strength of 7 T. The cylindrical samples were placed in an 8 mL glass vial, each filled with water, EtOH, THF, or n-Hep, and allowed to soak. After swelling was completed (usually after 24 h), the samples were placed in a transmit/receive volume coil with an inner diameter of 72 mm. Apparent diffusion coefficients (ADC) were gained from DW-MRI performed by using the following parameters: resolution 200 × 200 µm, image size 295 pixels × 90 pixels, slice thickness 1 mm, scan time 39 min, echo time (TE) 25.5 ms, and repetition time (TR) 5000 ms as well as for b-values of 0 s/mm^2^, 1000 s/mm^2^, and 2460 s/mm^2^ and three orthogonal diffusion directions. ADC calculation was based on the equation below.

(8) $$\frac{S}{S_{0}}=e^{-\gamma^{2}G^{2}\delta^{2}\left(∆-\frac{\delta }{3}\right)D}=e^{-\mathrm{bD}}$$

It was done for each voxel by the ISA Tool (Bruker BioSpin GmbH, Ettlingen, Germany) by fitting a curve of the signal intensity (with and without diffusion weighting) plotted over the b-value.

In addition, the diffusion front of water was visualized by placing the dried hydrogel cylinder in a water-filled crystallizing dish with a diameter of approximately 40 mm. The process was monitored for 23 h at the parameters mentioned above, except for TE = 35.0 ms and TR = 2500.0 ms. Among others, George and Whittaker described similar procedures in 2010 [32].

## 3. Results

### 3.1. Gravimetric Swelling Experiments

**Figure 2:**
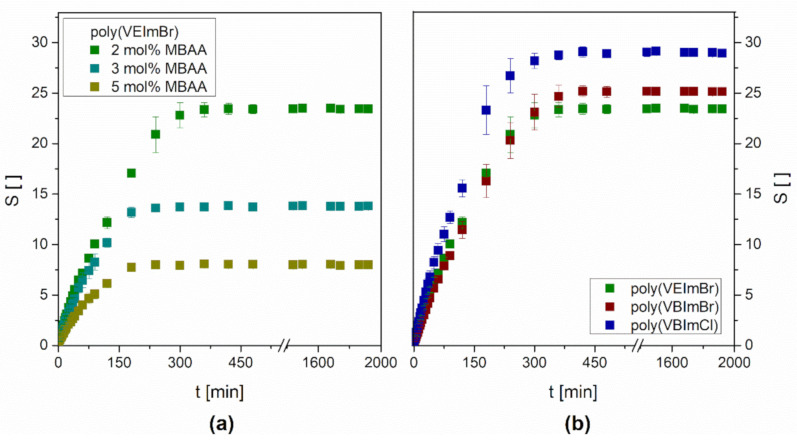
Water swelling kinetics (**a**) of poly(VEImBr) with different MBAA amounts (30 ± 1 °C; *n* = 3) and (**b**) of different monomers (30 ± 1 °C; *n* = 3; MBAA: 2 mol%).

A main characteristic of hydrogels is the ability to absorb large amounts of water with an increase in mass and volume without losing their shape. In principle, the swelling of polymer hydrogels consists of two separate transport steps. Initially, the respective solvent convects through the pores of the gel. Subsequently, the liquid diffuses between the struts of the polymer network causing a conformational change and an expansion of the polymer chains [36]. Besides the solvent motion and its interaction with the polymeric network, there are several parameters affecting the hydrogel swelling like the thermodynamic compatibility, the polymer relaxation time, the nature of the monomer, the crosslinker chain lengths, or the degree of crosslinking. Figure 2a shows the results of the water swelling tests at 30 ± 1 °C for poly(VEImBr) synthesized with different crosslinker concentrations of 2 mol%, 3 mol%, and 5 mol% MBAA. Independent of the crosslinker content, the mass of the hydrogels initially increased continuously over time until plateau formation occurred due to the setting of the swelling equilibrium. However, as the degree of crosslinking increased, the time required to reach swelling equilibrium decreased. Poly(VEImBr) crosslinked with 5 mol% MBAA was already in the equilibrium state after 240 min (4 h). Compared to that, 2 mol% crosslinked gels reached equilibrium state three hours later after 420 min. Nonetheless, the slopes of the graphs show that the hydrogels having a higher crosslinker level did not swell faster than lower crosslinked ones. The graph of poly(VEImBr) with 2 mol% MBAA rises significantly steeper than that of poly(VEImBr) with 5 mol% MBAA due to a higher initial swelling rate (Table 2). The reason for this was the setback of the swelling ratio plateau due to a lesser water adsorption by the stronger crosslinked hydrogel. This occurred because the interstices for liquid intercalation were gradually decreased the more tightly meshed the linkage was. The equilibrium swelling decreased by approximately 66% from 23.44 ± 0.39 to 8.00 ± 0.19 when the crosslinker amount was advanced from 2 mol% to 5 mol%.

The impact of the monomer was investigated by the usage of hydrogels based on two further monomers having longer chains and/or different counterions compared to VEImBr (Figure 2b). VBImBr contains a butyl group instead of an ethyl group while holding the same counterion. The prolonged alkyl side chain resulted in a slight increase of the swelling ratio by approximately 7% up to 25.16 ± 0.41 for poly(VBImBr). This observation seemed contradictory since the C4 chain is more hydrophobic than the C2 chain, resulting in a lower affinity to water. On the other hand, hydrogels with a butyl side chain have larger interstitial spaces for the incorporation of water. Obviously, this fact outweighs the impact of the increased hydrophobicity. In contrast to that, the variation of the counterion seemed to have a higher influence on the swelling. The swelling ratio of poly(VBImCl) rose to 29.02 ± 0.39 which was 16.5% higher than the swelling ratio of poly(VBImBr) and 23.8% higher compared to poly(VEImBr). A possible reason might be the stronger hydration of the smaller chloride anion (181 pm) compared to the bigger bromide anion (196 pm) [37]. Nevertheless, all hydrogels reached the equilibrium swelling plateau after 420 min. Additionally, the equilibrium water content (EWC) was calculated to represent the water that was absorbed by the equilibrium-swelled hydrogels in a quantitative manner (Table 3).

**Figure 3:**
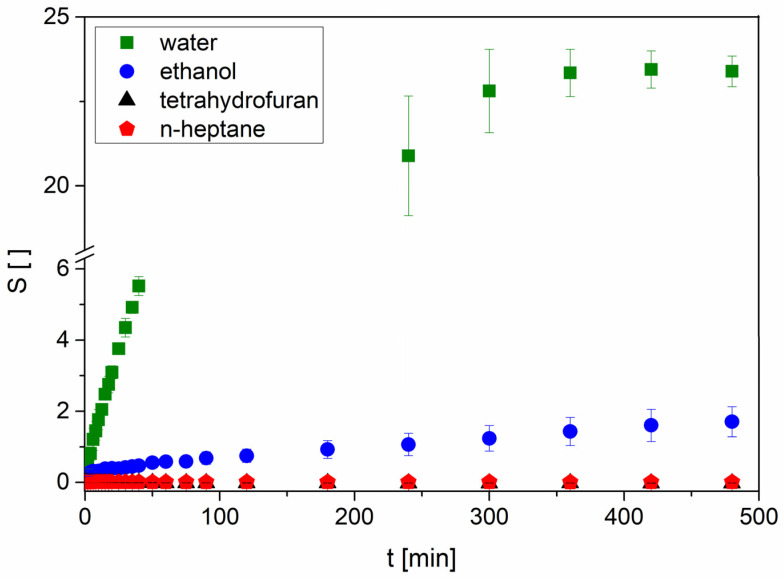
Swelling kinetics of poly(VEImBr) in different solvents (30 ± 1 °C; *n* = 3; MBAA: 2 mol%).

In medical applications, hydrogels are usually used in aqueous environments of different pH values containing a number of ions. For catalytical approaches, the behavior in other solvents is also very interesting. Therefore, we tested the swelling of poly(VEImBr) in EtOH, THF, and n-Hep as solvents with different polarities. Among these solvents, the strongest swelling of the gel was observed in EtOH with an equilibrium swelling of 7.35 ± 1.11 after 96 h (Figure 3). Apart from the lower swelling that was just one-third of the swelling in water, the process in EtOH was almost fourteen times slower due to the structure differences between both solvents. On the one hand, water is much smaller than EtOH so, it is easier for the molecule to enter the gel. On the other hand, there is a slight decrease in hydrophilicity. In previous works of Arndt et al., it has been shown that the swelling is decreasing due to a longer carbon chain of the respective solvent [38].

In *n*-Hep, the mass and volume of the gel cylinders remained fairly constant, whereas in THF a slight mass decrease was observed till an equilibrium plateau was reached. Apart from this, the gels in THF became very hard and incompressible over the measurement period. It is proposed that neither n-Hep nor THF diffuse into the gel. We suppose that the THF extracted the remaining unattached water and other components like unpolymerized monomer out of the hydrogel, leading to an additional decrease in mass compared to the dry initial state. In the case of *n*-Hep, the water most likely stayed in the gel because of the solvent polarity and the extremely low solubility of n-Hep in water. These general trends were also observed for poly(VBImBr) as well as poly(VBImCl) and poly(VEImBr) with an increased MBAA amount of 5 mol%.

The swelling kinetics studies of the experimental data showed a good agreement of the theoretical calculated equilibrium swelling with the experimental ones (Table 2). Even in the case of *n*-Hep and THF, where no swelling was observed, the values are parallel to each other. The calculation of the initial swelling rates shows no difference between 2 mol% and 3 mol% crosslinked poly(VEImBr) with 0.227(0) min^−1^ and 0.227(4) min^−1^, respectively. A light descent to 0.165 min^−1^ was observed when using 5 mol% MBAA for gel synthesis. In the case of poly(VBImBr), the initial swelling rate of 0.178 min^−1^ was smaller than the one of 2 mol% crosslinked poly(VEImBr), although the equilibrium swelling was higher. It is supposed that the longer and slightly hydrophobic side chain was responsible for the reduced swelling rate. All experiments in EtOH ended in a reduced initial swelling rate compared to water. Nevertheless, the difference between water and EtOH decreased due to the longer side chain. Furthermore, the swelling rate constant (k_s_) was calculated, which likely depended on two factors. It seems that k_s_ increased with a lower initial swelling rate combined with a reduced equilibrium swelling level. Among the experiments in water, 5 mol% crosslinked poly(VEImBr) showed the biggest value of 2.42 × 10^−3^ min^−1^. So, this hydrogel reached its equilibrium state much faster, probably due to its slightly reduced initial swelling rate and the significantly lower equilibrium swelling level. In contrast to that, k_s_ for 2 mol% poly(VEImBr) and poly(VBImCl) were reduced by the power of ten.

### 3.2. Diffusion Coefficients from Gravimetric Swelling Experiments

The diffusional exponent (*n*), which is the slope of the plot ln(*M_t_/M*_∞_) as a function of ln(*t*), is used to characterize the solute transport mechanism in hydrogels because the absorption process does not correspond to the classical theory of diffusion. In cylindrical hydrogels, values of 0.50 < *n* < 1 correspond to non-Fickian or anomalous diffusion. If the transport mechanism is relaxation-controlled, a Case-II-diffusion and *n* = 1 can be observed. Fickian-type transport occurs when the polymer chain relaxation rate is greater than the water penetration rate in the gel. In most cases, *n* = 0.45–0.50 implies an impeccable Fickian-type process, but some articles also report *n* < 0.50 [31,39]. In the case of pure Fickian-type diffusion, the transport process is determined exclusively by diffusive currents, and it is independent of other physical effects such as swelling of the gel matrix. However, these gels showed strong swelling in polar protic solvents like water. In this case, diffusion is superimposed on time-dependent swelling and deviations from Fickian diffusion behavior occur [32,40]. This is also reflected in the diffusional exponents for the water diffusion in the cylindrical pILs-based hydrogels. The values varied between 0.668 for 5 mol% crosslinked poly(VEImBr) and 0.837 for poly(VBImBr), indicating non-Fickian behavior (Table 4). The calculated diffusion coefficients (D) of water are in the range of 10^−10^ m^2^ s^−1^ and correspond to the values reported by Bajpai et al. for the diffusion of water in poly(acrylamide-*co*-sodium acrylate) hydrogels [31]. In a number of cases, the coefficients for cylindrical gels are usually in the range of 10^−11^ to 10^−12^ m^2^ s^−1^ [31,41,42]. The higher diffusion coefficients indicate a faster solvent transport into the polymer network. They were caused by the osmotic pressure of the gel, which must be overcome permanently by the solvent during the swelling procedure. Consequently, the osmotic pressure is also responsible for the deviation of the transport mechanism from the Fickian behavior [43]. In this method, there was nearly no difference observed concerning the coefficients calculated for 2 mol% poly(VEImBr) and poly(VBImBr). A moderate increase concerning the coefficient was observed for poly(VBImCl) with 7.65 ± 0.92 × 10^−10^ m^2^ s^−1^ compared to 2 mol% crosslinked poly(VBImBr) with 6.67 ± 1.15 × 10^−10^ m^2^ s^−1^. A possible explanation for the higher coefficient could be that the more water is stored between the network struts, the more freely the water molecules can move inside the gel network. Thus, the measured values can change in the direction of the self-diffusion coefficient of water, which is 2.59 × 10^−9^ m^2^ s^−1^ at 30 °C [44]. On the contrary, increasing the crosslinker content in poly(VEImBr) had no significant influence. This can possibly be explained by the fact that the tighter linkage resulted in a mesh size that was still large enough for the entry of the respective solvent molecules [31]. The only slightly minimized initial swelling rate also supports this thesis. For EtOH, the determined n values were comparatively lower, but they also indicate non-Fickian diffusion. Only 2 mol% crosslinked poly(VEImBr) with *n* = 0.398 implies a transport mechanism according to Fick. Compared to the values in water, the diffusion coefficients in EtOH are reduced by the power of ten. Since the self-diffusion coefficient of EtOH (1.08 × 10^−9^ m^2^ s^−1^) is also nearly reduced by the power of ten compared to the coefficient of water, the calculated values reflect the ratio of the two pure solvents [45]. Since no mass increase was observed in *n*-Hep and in THF, diffusion coefficients could not be determined using this method.

### 3.3. Sorption Curves

**Figure 4:**
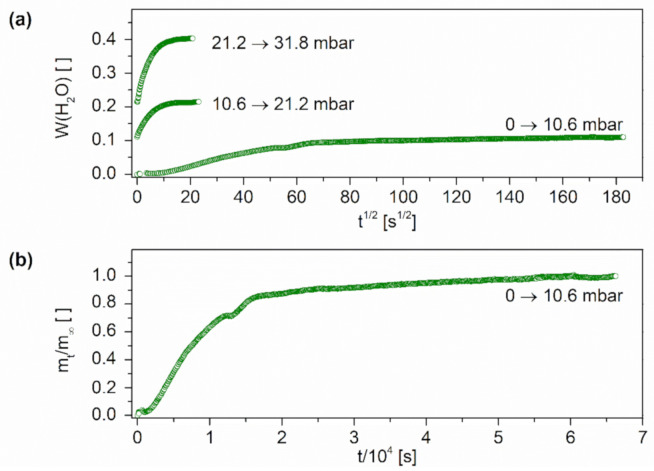
Interval sorption curves of poly(VEImBr) and water (**a**) for different pressure steps up to 31.8 ± 0.2 mbar; (**b**) plotted as the relative mass increase over time for the first pressure step up to 10.6 mbar (30 ± 1 °C; MBAA: 2 mol%).

Sorption curves usually characterize the sorption behavior of the sample at different partial pressures of the respective solvent. In the conducted experiments, we set 25%, 50%, and 75% of the total solvent partial pressure. In the case of water, the partial pressure corresponds to the relative humidity. Figure 4a shows the recorded sorption curves of water in a poly(VEImBr) film (2 mol% MBAA) at 30 °C and 10.6 mbar (25% RH), 21.6 mbar (50% RH), as well as 31.8 mbar (75% RH).

For graphical representation, W was plotted as the mass of absorbed water relative to the mass of the dry polymer sample versus the square root of the time. The new step always started with the equilibrium mass of the previous stage at the time t = 0 s. In the first sorption interval from 0 to 10.6 mbar, the recorded curve exhibits a slight sigmoidal shape, which indicates non-Fickian behavior. For the following pressure steps at 50% RH and 75% RH, a rather Fickian-type shape was obtained. Characteristically, Fickian-type curves show initial linear increasing that flattens out and finally settles at an equilibrium value. When plotting the pressure jump intercepts versus time t, a nearly linear growth of the sample mass was observed over the measurement period (Figure 4b). This pattern is characteristic of Case-II-diffusion, which often occurs with larger concentration jumps (∆W > 0.1 g/g) and is associated with relatively steep concentration fronts within the sample. Characteristic of these sharp fronts is a large difference between the diffusion coefficients in the swollen and unswollen regions of the gel [34]. A visualization of the diffusion front could provide additional information concerning the concentration profiles into the gel. Generally, a deviation from Fickian-type behavior is observed for measurements outside the glass transition temperature. Especially for experiments below this temperature, a deviant behavior is observed. Another explanation is the strong swelling of the hydrogels. Within swelling, solvents like water penetrate into the polymer network, forcing the polymer molecules to rearrange themselves. Depending on the structure and condition of the polymer, the reorientation runs at different speeds and a tension between the polymer chains occurs. As a result, there is an increased pressure on the water molecules, which limits the uptake of new solvent molecules into the polymer matrix. The time-dependent swelling finally leads to a relaxation of the chains combined with a reduction in pressure, whereby new molecules can be taken up. Furthermore, so-called transverse forces are formed between the solvent-poor and the solvent-rich regions of the sample. All in all, both phenomena, viscoelastic volume swelling and swelling transverse forces, usually occur together and are difficult to separate from each other [46]. Similar sorption curves were obtained for the 5 mol% crosslinked poly(VEImBr) as well as for poly(VBImCl) and poly(VBImBr) with water. Logically, all curves showed the steepest increase during the last sorption step up to 31.8 mbar because of the highest solvent content in the surrounding atmosphere. In all gels, the mass of the absorbed water was nearly doubled from step to step. Concerning the equilibrium swelling at the different solvent pressures, poly(VEImBr) achieved 0.111 at 25% RH, 0.215 at 50% RH, and 0.403 at 75% RH. So it swelled almost twice as much as poly(VBImBr) with 0.057 at 25% RH, 0.122 at 50%, RH and 0.257 at 75% RH. Similar to the previous experiments, poly(VBImCl) showed the strongest swelling with 0.103 at 25% RH, 0.218 at 50% RH, and 0.482 at 75% RH. The sorption measurements also showed a difference between the poly(VEImBr) hydrogels having varied MBAA contents. In the 5 mol% hydrogel the uptake was reduced by 11%. As expected, the sorption curves of the experiments with EtOH also showed a deviation from Fickian-type diffusion behavior. Furthermore, the gels absorbed less EtOH while having significantly longer swelling times to reach sorption equilibrium compared to the experiments with water. A mass loss was detected for *n*-Hep and THF. In *n*-Hep, the sample mass fluctuated and finally decreased minimally by 1.7 mg within the measurement period of 24 h. This phenomenon may be explained by the buoyancy within the measuring tube caused by the upward flowing solvent vapor. The hydrogel sample was placed in a glass basket connected to the balance by a spring. Thus, the bottom of the glass vessel was fully exposed to the solvent vapor flowing from the bottom to the top. In contrast to that, the mass decreased stepwise when measuring with THF. This observation supports our thesis that THF probably draws the unbound water out of the hydrogel, resulting in weight reduction.

### 3.4. Water Diffusion Coefficients from Gravimetric Sorption Experiments

Sorption measurements were performed to determine the Fick’s diffusion coefficient of water and other solvents into the sample. The coefficient describes how fast the solvent molecules move in the solvent gradient of the mixture and depends on the relative humidity of the solvent. Since the relative humidity is influencing the solvent concentration in equilibrium, the calculated diffusion coefficient becomes dependent on the solvent concentration and thus overrides Fick’s definition as a proportionality constant. Consequently, relative statements about the diffusion rate as a function of relative humidity are obtained from the calculated coefficients. As expected, D varied depending on the surrounding water partial pressure RH in the apparatus and was lowest at 25% RH (Table 5). It is noticeable that the diffusion of the molecules was most pronounced at a relative humidity of 50% and decreased again at 75% RH. Apparently, the driving force was no longer as high at 75% RH due to lower concentration gradients. Strictly speaking, differences in the chemical potentials were the cause of this observation. However, the Fick’s model used here is a concentration-based system, which does not take into account the influence of the potential differences. At the last sorption interval there was already a certain number of water molecules in the gel, leading to a reduction of the diffusion compared to the condition at 50% RH. Likely, this was a hydrogel-specific property caused by the lattice structure. Linear polymers are able to unwind their chains and therefore do not exhibit these effects. In contrast to that, the space is limited in the networks of the hydrogels. So, there is just a defined number of molecules that are allowed to penetrate the structure. However, at 25% RH there was still a strong concentration gradient, but the absorption capacity of the water molecules was limited by the gel swelling.

### 3.5. DW-MRI Measurements

**Figure 5:**
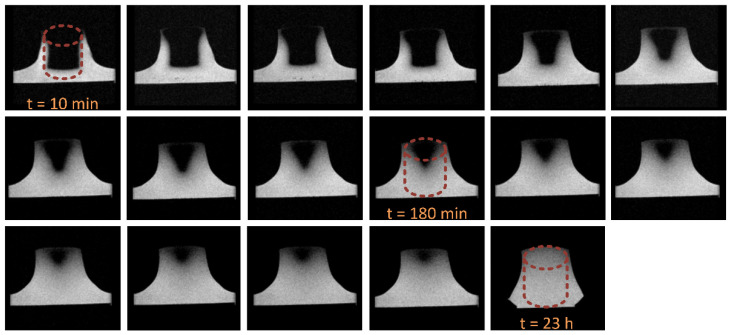
T2-weighted visualization of the water diffusion front in poly(VEImBr) (21.5 ± 2 °C; TR: 2500.0; TE: 35.0; MBAA: 2 mol%).

DW-MRI as an imaging technique has developed into a decisive method in medical diagnostics over the last 30 years. It enables the measurement and visualization of solvent diffusion, typically water, that is caused by Brownian motion. This type of molecular movement is called self-diffusion and does not require concentration gradients because it relies solely on the thermal energy of the solvent molecules. However, DW-MRI does not measure free diffusion because molecular motion is restricted by structural obstacles in the corresponding tissues [47,48]. Compared to gravimetric studies where the molecular diffusion is measured by means of macroscopic changes, MRI allows a deeper insight into the diffusion procedure providing direct information about the water distribution within the polymer [32]. The visualization of the water diffusion front in 2 mol% crosslinked poly(VEImBr) is shown in Figure 5. For better clarity, the outline of the cylindrical specimen has been added to the graph in the form of red dashed lines. During the measurement, the hydrogel cylinder stood in a layer of water and after 10 min the penetration of water could already be observed in the lower area of the gel as well as on the lateral outer surfaces. Over time more water diffused into the sample, causing the gel to swell from the bottom and laterally upward. The diffusion front could be observed very well during the whole process, which indicated strong concentration differences in the hydrogel regions. This supports the thesis of Case-II-diffusion from the sorption measurements above. After 23 h the measurement was stopped because the entire gel body was penetrated with water. In contrast to the swelling measurements where complete swelling was finished after 420 min, here the hydrogel required much longer because of the experimental setup. In this case, the gel was placed in a thin layer of water instead of being completely enclosed.

**Figure 6:**
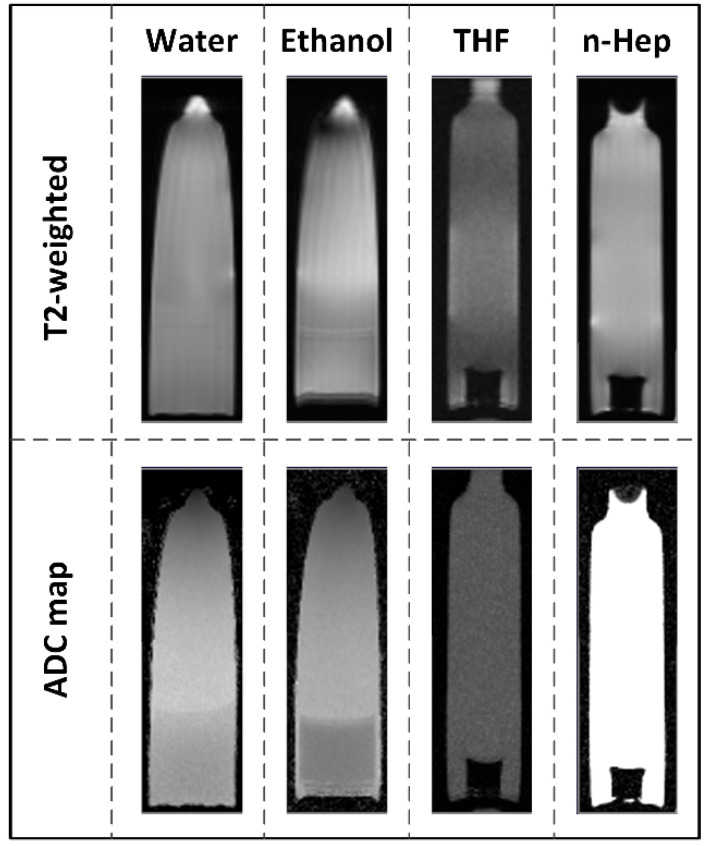
T2-weighted visualization and ADC map of the solvent diffusion in poly(VEImBr) (21.5 ± 2 °C; TR: 2500.0; TE: 35.0; MBAA: 2 mol%).

Due to the limited molecular movement in tissues, the diffusion coefficient measured via DW-MRI is called the apparent diffusion coefficient (ADC) and is dependent on the direction and length of the diffusion. However, if the size of the tested structure is known, the measurement time can be adjusted and the dependence on the diffusion length can be neglected. All measured ADCs are summarized as the results of method 3 in Table 6. Although the measurements were implemented at lower temperatures compared to the gravimetric methods, the determined coefficients are bigger than the ones measured by the gravimetric studies. In water, a slightly decreased ADC was observed at higher degrees of swelling. Among the tested monomers, poly(VEImBr) showed the lowest level at equilibrium swelling, but achieved the highest ADC of 16.58 ± 0.59 × 10^−10^ m^2^ s^−1^ in the MRI experiments. The ADC of poly(VBImBr) was 16.30 ± 0.47 × 10^−10^ m^2^ s^−1^ while the strongest swelling poly(VBImCl) hydrogel achieved 15.80 ± 0.55 × 10^−10^ m^2^ s^−1^. Nevertheless, as expected the ADCs of the hydrogels were smaller than the measured value of 19.64 ± 0.59 × 10^−10^ m^2^ s^−1^ for the self-diffusion of pure water. A lower coefficient of 15.90 ± 0.53 × 10^−10^ m^2^ s^−1^ was also detected for poly(VEImBr) with 5 mol% MBAA. Presumably an increased crosslinker concentration resulted in a stronger inhibition of the molecular diffusion due to the additional linkages. Compared to water, the diffusion of ethanol in the 2 mol% crosslinked hydrogels was again slower. The coefficients of ethanol varied between 6.51 ± 0.23 × 10^−10^ m^2^ s^−1^ in the case of poly(VEImBr) and 6.65 ± 0.47 × 10^−10^ m^2^ s^−1^ for poly(VBImBr). The large ADC of 7.08 ± 0.62 × 10^−10^ m^2^ s^−1^ for the 5 mol% poly(VEImBr) appears contradictory, but this and the relatively high deviation can be explained by the tearing of the sample during the swelling process. Moreover, the ADCs for the experiments with 2 mol% crosslinked poly(VEImBr) in *n*-Hep or THF should be determined as well. The MRI scans support the assumption that in both cases nearly no solvent diffused into the hydrogels. In these samples, only very small ADCs with large error margins were obtained, such as 7.15 ± 15.20 × 10^−11^ m^2^ s^−1^ for THF. This coefficient may correspond to the background noise, which showed a value of 9.01 ± 17.27 × 10^−11^ m^2^ s^−1^. The same was observed with *n*-Hep, where the measured coefficient of 7.98 ± 17.80 × 10^−11^ m^2^ s^−1^ was in the same scale of the background noise (6.50 ± 14.97 × 10^−11^ m^2^ s^−1^). In these cases, the signal was too weak to determine a reliable ADC. This is supported by the acquired MRI images, which are shown in Figure 6. In the case of water and ethanol, the gel sample and the pure solvent have nearly the same coloration. Accordingly, diffusion of the solvent into the hydrogel had occurred. In THF and *n*-Hep, the non-swollen hydrogel is shown in black and stands out clearly from the light gray color of the pure solvent. The measured self-diffusion coefficients of *n*-Hep and THF were much higher with 2.85 ± 0.17 × 10^−9^ m^2^ s^−1^ and 2.50 ± 0.27 × 10^−9^ m^2^ s^−1^, respectively.

## 4. Discussion

From the gravimetric swelling experiments, it is evident that the tested pILs-based hydrogels have a high affinity to polar protic solvents. In general, the strongest mass increase was observed in the experiments with water. For instance, 2 mol% crosslinked poly(VEImBr) showed a three times higher equilibrium swelling level in water (23.44 ± 0.39) compared to EtOH (7.45 ± 1.02). The following order was achieved for the different monomers with regard to their equilibrium swelling in water and EtOH: poly(VEImBr) < poly(VBImBr) < poly(VBImCl). Increasing the crosslinker content of poly(VEImBr) from 2 mol% to 5 mol% resulted in a reduced mass increase of 8.00 ± 0.19. In n-Hep and THF, the mass remained unchanged or even decreased slightly. The investigation of the diffusion mode in the hydrogels showed a deviation from Fickan-type diffusion both in the gravimetric swelling experiments and in the case of the sorption curves from the measurements with the magnetic suspension balance. The exact classification of the diffusion type in hydrogels often proves to be very difficult as soon as the diffusion deviates from Fickian-type behavior [40]. In this study, the calculations of the *n*-values from the data of the swelling tests indicated an abnormal diffusion while the shape of the sorption curves argued for a Case-II-diffusion. We tried to support the results with a visualization of the diffusion front via DW-MRI and found sharp concentration fronts that also point in favor of Case-II-diffusion. The diffusion coefficients were determined by three different methods and the results for water and EtOH are summarized in Table 6. Since no mass increase was recorded for the samples in *n*-Hep or THF using the gravimetric methods, diffusion coefficients could not be calculated. In general, it is assumed that these solvents either do not diffuse or diffuse minimally into the gel samples. In THF, a mass loss was observed, which indicates that the solvent draws the unbound water out of the gel. This is an interesting approach for the application of a co-drying agent. For water, nearly all coefficients are in the range of 10^−10^ to 10^−11^ m^2^ s^−1^. The biggest values were obtained from the DW-MRI measurements. This trend was also observed with EtOH, with coefficients about one power of ten smaller than using water. It noteworthy that depending on the measurement method the values of the same sample may vary. An important point is the accuracy of the measurement method and which parameters are used to determine the diffusion coefficient. Gravimetric swelling measurements are one of the most common methods to investigate diffusion processes. Unfortunately, it also seems to be the most error-prone of all the methods tested here due to the experimental setup and implementation. DW-MRI offers the possibility to follow the diffusion processes in the hydrogel itself and to determine the coefficients directly in the gel, whereas the sorption measurements use macroscopic changes to evaluate the microscopic processes. However, in the case of the magnetic suspension balance, it runs in an experimental setup that makes external influences almost negligible and thus minimizes errors. All in all, these results provide interesting information about diffusion in pILs-based hydrogels. Possible effects of significantly longer alkyl chains as well as the change of other structural components are also interesting topics for future work. Thus, the processes occurring in polyelectrolyte-based hydrogels will be investigated in subsequent work.

## Figures and Tables

**Table 1:** Hydrogel composition with different crosslinker contents.

Component	Crosslinker Content		
	2 mol%	3 mol%	5 mol%
**IL monomer**	0.300 g VEImBr/0.341 g VBImBr/0.276 g VBImCl		
**Ultrapure water**	432 µL	312 µL	65 µL
**MBAA solution (2% *w*/*w*)**	232 µL	352 µL	599 µL
**APS solution (10% *w*/*w*)**	30 µL		
**TEMED**	6 µL		

**Table 2:** Swelling kinetics studies of different hydrogels.

Hydrogel MBAA Amount	Solvent	Experimental Equilibrium Swelling Seq, exp ( )	Theoretical Equilibrium Swelling Seq, theo ( )	Initial Swelling Rate (min^−1^)	Swelling Rate Constant ks (min^−1^)
**Poly(VEImBr) 2 mol%**	Water	23.44 ± 0.39	25.13	0.227	3.60 × 10^−4^
	EtOH	7.45 ± 1.02	8.14	0.010	1.58 × 10^−4^
	THF	−0.02 a	−0.02	n.d. *	n.d. *
	n-Hep	0.01 b	0.01	n.d. *	n.d. *
**Poly(VEImBr) 3 mol%**	Water	13.80 ± 0.25	14.39	0.227	1.10 × 10^−3^
**Poly(VEImBr) 5 mol%**	Water	8.00 ± 0.19	8.25	0.165	2.42 × 10^−3^
	EtOH	2.56 ± 0.12	2.79	0.005	5.81 × 10^−4^
	THF	−0.02 a	−0.02	n.d. *	n.d. *
	n-Hep	0.01 b	0.01	n.d. *	n.d. *
**Poly(VBImBr) 2 mol%**	Water	25.16 ± 0.41	27.70	0.178	2.32 × 10^−4^
	EtOH	10.61 ± 0.62	11.67	0.021	1.53 × 10^−4^
	THF	−0.01 a	−0.01	n.d. *	n.d. *
**Poly(VBImCl) 2 mol%**	Water	29.02 ± 0.39	31.15	0.277	2.86 × 10^−4^
	EtOH	13.51 ± 0.40	14.62	0.039	1.80 × 10^−4^
	THF	−0.01 a	−0.01	n.d. *	n.d. *

* n.d.—not determined due to the lack of swelling; ^a^ the error was <0.005; ^b^ the measured values were in the order of magnitude of the error due to insignificant changes in weight.

**Table 3:** Equilibrium water contents of pILs-based hydrogels.

Hydrogel	MBAA Amount	Equilibrium Termination	Equilibrium Water Contents (EWC)
Poly(VEImBr)	2%	420 min	0.959 ± 0.001
	3%	360 min	0.932 ± 0.001
	5%	240 min	0.889 ± 0.003
Poly(VBImBr)	2%	420 min	0.962 ± 0.001
Poly(VBImCl)	2%	420 min	0.967 ± 0.001

**Table 4:** Diffusional exponent *n* and diffusion coefficient D determined by gravimetric swelling studies at 30 °C.

Solvent	Diffusion Parameter	Poly(VEImBr) 2 mol%	Poly(VEImBr) 5 mol%	Poly(VBImBr) 2 mol%	Poly(VBImCl) 2 mol%
**Water**	**n**	0.800	0.668	0.837	0.830
	**D × 10^10^ (m^2^ s^−1^)**	6.70 ± 0.85	6.95 ± 0.40	6.67 ± 1.15	7.65 ± 0.92
**EtOH**	**N**	0.398	0.572	0.609	0.634
	**D × 10^10^ (m^2^ s^−1^)**	0.37 ± 0.11	0.62 ± 0.23	0.61 ± 0.23	1.07 ± 0.09

**Table 5:** Water diffusion coefficients in the hydrogels for different relative humidities at 30 °C.

Hydrogel	MBAA Amount	Diffusion Coefficient D × 10^10^ (m^2^ s^−1^) at Different Water RH		
		25%	50%	75%
**Poly(VEImBr)**	2 mol%	0.130	0.560	0.553
	5 mol%	0.110	0.322	0.262
**Poly(VBImBr)**	2 mol%	0.113	0.236	0.195
**Poly(VBImCl)**	2 mol%	0.035	0.144	0.097

**Table 6:** Summary of all diffusion coefficients measured using different methods.

Solvent	Method *	(Apparent) Diffusion Coefficient D/ADC × 10^10^ (m^2^ s^−1^) For			
		Poly(VEImBr) 2 mol%	Poly(VEImBr) 5 mol%	Poly(VBImBr) 2 mol%	Poly(VBImCl) 2 mol%
**H_2_O**	**1**	6.70 ± 0.85	6.95 ± 0.40	6.67 ± 1.15	7.65 ± 0.92
	**2**	0.553	0.262	0.195	0.097
	**3**	16.58 ± 0.59	15.90 ± 0.53	16.30 ± 0.47	15.80 ± 0.55
**EtOH**	**1**	0.37 ± 0.11	0.62 ± 0.23	0.61 ± 0.23	1.07 ± 0.09
	**2**	0.035	0.025	0.136	0.708
	**3**	6.51 ± 0.23	7.08 ± 0.62	6.65 ± 0.47	6.63 ± 0.25

* 1—gravimetric swelling experiments (30 ± 1 °C); 2—sorption experiments (30 ± 1 °C, *p*(water) = 31.8 (75% RH)); 3—DW-MRI measurements (21.5 ± 2 °C).

## Data Availability

The data presented in this study are available upon reasonable request from the corresponding author.

## Supplementary Materials

The following are available online at https://www.mdpi.com/article/10.3390/polym13111834/s1, Figure S1: ^1^H-NMR of VEImBr [300 MHz]; Figure S2: ^1^H-NMR of VBImCl [300 MHz]; Figure S3: ^1^H-NMR of VBImBr [300 MHz].
