# Supplementary material for: Swelling and Diffusion in Polymerized Ionic Liquids-Based Hydrogels
Source: Polymers (Basel). 2021 Jun 1;13(11):1834. doi: 10.3390/polym13111834 (PMC8199506; doi:10.3390/polym13111834)
Supplement: Supplementary file 1 [file polymers-13-01834-s001.zip › polymers-1239810-supplementary.pdf]

polymers

MDPI

Article

Swelling and Diffusion in Polymerized Ionic Liquids-Based Hydrogels

Ann Jastram [1], Tobias Lindner [2], Christian Luebbert [3], Gabriele Sadowski [4] and Udo Kragl [1,5,*]

[1] Institute of Chemistry, Industrial Chemistry, University of Rostock, Albert-Einstein-Str. 3a, 18059 Rostock, Germany; ann.jastram@uni-rostock.de
[2] Core Facility Multimodal Small Animal Imaging, Rostock University Medical Center, Schillingallee 69a, 18057 Rostock, Germany; tobias.lindner@med.uni-rostock.de
[3] amofor GmbH, Otto-Hahn-Str. 15, 44227 Dortmund, Germany; luebbert@amofor.de
[4] Department of Biochemical and Chemical Engineering, Laboratory of Thermodynamics, TU Dortmund University, Emil-Figge-Str. 70, 44227 Dortmund, Germany; gabriele.sadowski@tu-dortmund.de
[5] Department Life, Light & Matter, Faculty for Interdisciplinary Research, University of Rostock, Albert-Einstein-Str. 25, 18059, Rostock, Germany
* Correspondence: udo.kragl@uni-rostock.de; Tel.: +49-381-498-6450

Citation: Jastram, A.; Lindner, T.; Luebbert, C.; Sadowski, G.; Kragl, U. Swelling and Diffusion in Polymerized Ionic Liquids-Based Hydrogels. *Polymers* **2021**, *13*, 1834. https://doi.org/10.3390/polym13111834

Academic Editor: Ciprian Iacob

Received: 13 May 2021
Accepted: 26 May 2021
Published: 1 May 2021

Publisher's Note: MDPI stays neutral with regard to jurisdictional claims in published maps and institutional affiliations.

Copyright: © 2021 by the authors. Licensee MDPI, Basel, Switzerland. This article is an open access article distributed under the terms and conditions of the Creative Commons Attribution (CC BY) license (http://creativecommons.org/licenses/by/4.0/).

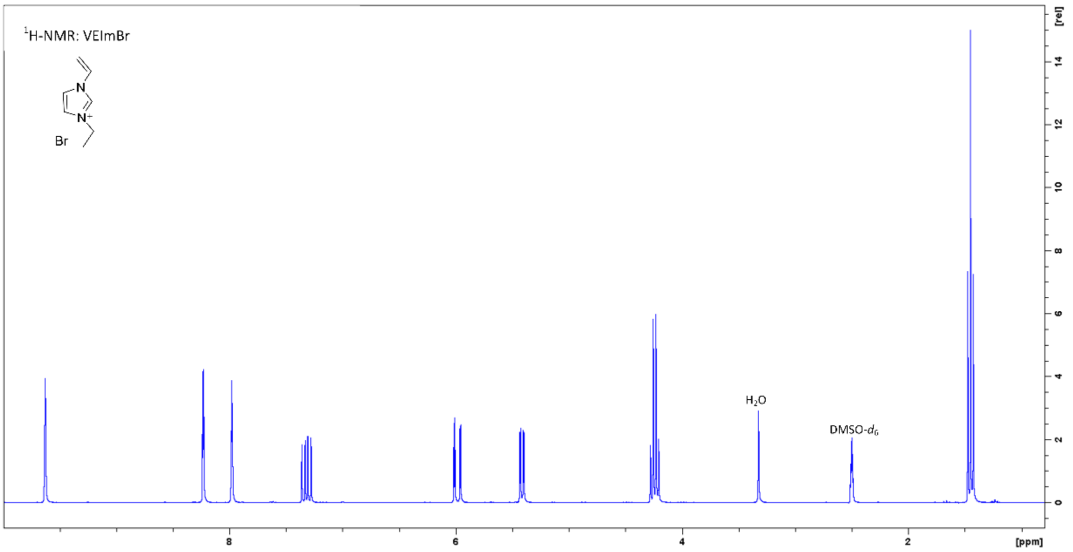

Figure S1. ^{1}H-NMR of VEImBr [300 MHz].

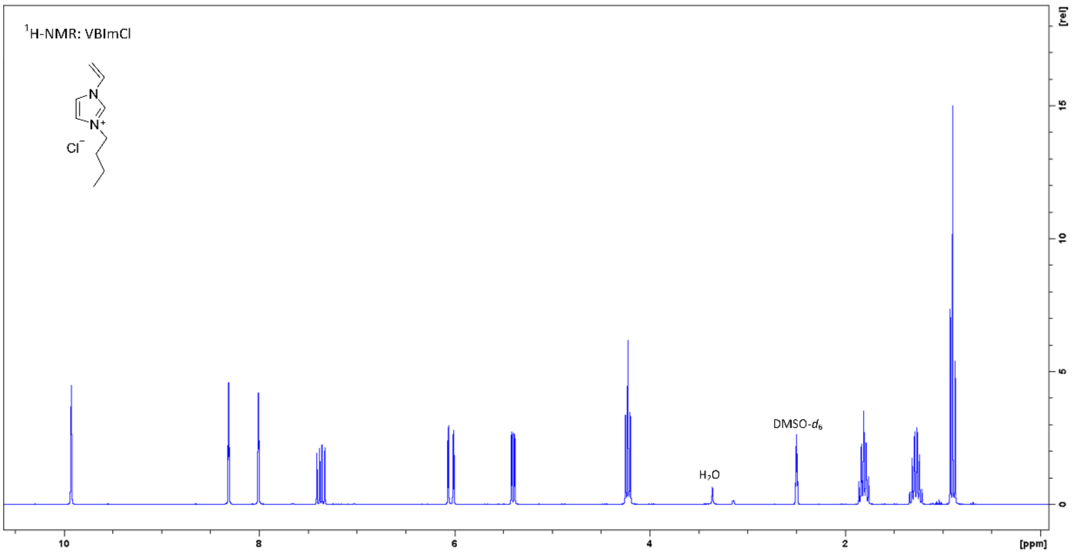

Figure S2. ^{1}H-NMR of VBImCl [300 MHz].

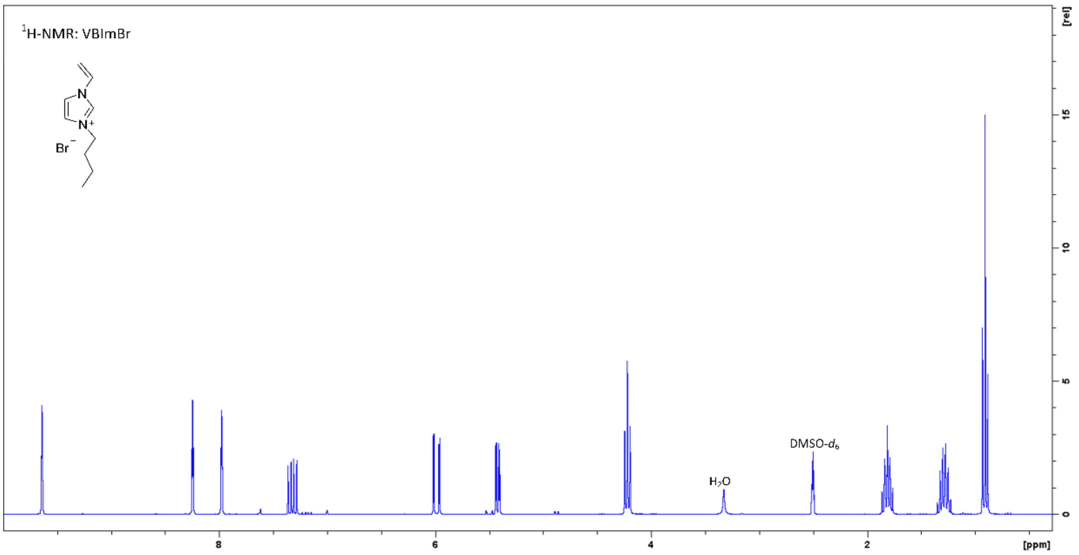

Figure S3. ^{1}H-NMR of VBImBr [300 MHz].
